# Functional outcomes and healthcare utilization trends in postsurgical and nonsurgical patients following high-frequency (10 kHz) spinal cord stimulation therapy

**DOI:** 10.3389/fpain.2024.1451284

**Published:** 2024-11-11

**Authors:** Vinicius Tieppo Francio, Logan Leavitt, John Alm, Daniel Mok, Byung-Jo Victor Yoon, Niaman Nazir, Christopher M. Lam, Usman Latif, Timothy Sowder, Edward Braun, Andrew Sack, Talal W. Khan, Dawood Sayed

**Affiliations:** ^1^ Department of Anesthesiology and Pain Medicine, The University of Kansas Medical Center, Kansas City, KS, United States; ^2^ Department of Physical Medicine and Rehabilitation, The University of Kansas Medical Center, Kansas City, KS, United States; ^3^ Department of Population Health, The University of Kansas Medical Center, Kansas City, KS, United States

**Keywords:** spinal cord stimulation, chronic low back pain, failed back surgery syndrome, persistent spinal pain syndrome, non-surgical refractory back pain, healthcare utilization, disability

## Abstract

**Introduction:**

Chronic low back pain (CLBP) is the leading cause of disability in the United States and is associated with a steadily increasing burden of healthcare expenditures. Given this trend, it is essential to evaluate interventions aimed at reducing disability and optimizing healthcare utilization (HCU) in affected populations. This study investigates the impact of prior spinal surgery on functional outcomes and HCU patterns following high-frequency (10 kHz) spinal cord stimulation (SCS) therapy.

**Methods:**

This retrospective observational study included 160 subjects who underwent implantation of a 10 kHz SCS device. Participants were divided into surgical and non-surgical cohorts for comparative analysis. Pain relief was assessed using the Numeric Rating Scale (NRS), while disability levels were evaluated using the Oswestry Disability Index (ODI). HCU was examined through the frequency of emergency department (ED) visits, outpatient visits for interventional pain procedures, and opioid consumption measured in morphine milliequivalents (MME).

**Results:**

No statistically significant differences were observed between the surgical and non-surgical groups regarding pain relief and disability outcomes. Additionally, ED visits and outpatient visits for interventional pain procedures did not show significant differences between the two cohorts.

**Discussion:**

This study represents the first comparative analysis of pain, disability, and HCU trends between surgical and non-surgical populations following 10 kHz SCS therapy. The results suggest that prior spinal surgery may not substantially affect the efficacy of 10 kHz SCS therapy in terms of pain relief, disability reduction, or HCU patterns.

## Introduction

In the United States, chronic low back pain (CLBP) stands as the leading cause of disability, with its indirect healthcare costs estimated as high as USD 624.8 billion (1–3). Moreover, the global burden of CLBP is projected to escalate further in the forthcoming years with estimated healthcare costs that may reach up to USD 20 billion (4, 5). This economic burden is influenced by various factors, including frequent outpatient visits, diagnostic tests, prescription medications, rehabilitation services, injections, and surgery (4). As such, a great opportunity to lessen pain, disability, disease burden and reduce direct and indirect health care utilization (HCU) exists by targeting this population (6).

Spinal cord stimulation (SCS) has been an established treatment with high-quality level I evidence from multiple pre-clinical and prospective studies, randomized controlled trials (RCT), and supported by multiple society guidelines for the treatment of CLBP (7–12). Historically, SCS was utilized for complex regional pain syndrome (CRPS) and failed back surgery syndrome (FBSS), recently renamed persistent spinal pain syndrome (PSPS type II) (13–16). PSPS type II is defined as chronic axial back pain and/or radicular pain after spinal surgery, and it is estimated to affect 10%–40% of patients following large surgical intervention, while PSPS type 1 is chronic axial back pain and/or radicular pain without history of prior surgery (17). The etiology of PSPS type II is multifactorial in nature and may arise directly from surgical complications, tissue manipulation, recurrence of pathology, incomplete resolution of symptoms, or indirectly from biomechanical changes post-surgery. Patients may present with components of neuropathic, nociceptive, nociplastic pain or a mixed pain syndrome, which makes it a challenging condition to treat (15, 17). Treatment options vary from conventional medical management (CMM) with pharmacological management, physical therapy, spinal injections, neuromodulation and re-operation. In carefully selected patients with PSPS type II who have failed conservative therapies and responded well to a SCS trial, SCS therapy is well-established as an effective treatment with a moderate level of evidence (10, 17, 18).

Recent advancements in neuromodulation technology have broadened the utility of SCS therapy as a Food and Drug Administration (FDA) approved treatment option beyond traditional indications to include painful diabetic neuropathy (PDN) and nonsurgical refractory chronic low back pain (NSRBP) (12, 19, 20). Treatment for chronic axial low back pain primarily includes the CMM (21, 22). However, there is a subset of patients that do not respond to CMM, and these are classified as NSRBP patients (19). The definition of NSRBP is broad and not specific to a particular etiology, however it is thought to have primarily neuropathic features (18, 19). NSRBP patients present with long standing chronic axial low back pain that does not respond to CMM and without a history of spine surgery, and these or are not candidates for spine surgery following evaluation by a spine surgeon (18). In such patients, SCS can be considered as a treatment option and recent studies have demonstrated that the addition of SCS to CMM may offer significant improvement in pain, function, quality of life, and reduced opioid use long-term (12, 23).

Distinctive patient factors may impact outcomes in neuromodulation, and despite recent studies reporting functional outcomes and health care cost reduction in subjects who underwent SCS therapy, no studies have provided a head-to-head comparison of outcomes between surgical and nonsurgical groups Therefore, this study aimed to analyze if a history of spinal surgery affects functional outcomes and healthcare utilization trends following 10 kHz SCS therapy.

## Materials & methods

### Participants

The study enrolled 160 participants from a single-center. Participants were adults of at least 18 years of age who experienced CLBP refractory to CMM, stemming from etiologies such as PSPS and NSRBP. Subjects were selected between August 1, 2019, and December 31, 2021. Inclusion criteria were not restrictive in terms of race, gender, socioeconomic status, healthcare insurance coverage, or any other demographic factors. Exclusion criteria included failure to meet the aforementioned requirements, and absolute contraindications to percutaneous placement such as uncontrolled coagulopathy, severe thrombocytopenia, active infection, or prior implantation of neuromodulation devices using waveforms other than 10 kHz. All participants provided informed consent for the procedure and had at least 12 months of (pre and post intervention) data for analysis. Participants were stratified into two groups based on their history of lumbar spinal surgery: group A (surgical history) included 81 subjects, while Group B (nonsurgical history) included 79 participants. Subjects included were those who demonstrated >50% pain reduction during a 10 kHz SCS trial, which subsequently underwent permanent SCS implantation with anatomical lead placement. The SCS trial and implantation were conducted utilizing percutaneously placed SCS leads, avoiding the need for an invasive procedure such as laminotomy. The SCS system used was the Omnia device manufactured by Nevro Corp, currently the sole provider of 10 kHz waveform capability in the United States. Post-implantation, subjects underwent a follow-up visit within seven days for wound assessment. Subsequent follow-ups were conducted at 3 weeks for further wound evaluation, and thereafter at 6 weeks, 3 months, and as required. Stimulation settings/parameters were adjusted via a remote control by the patient and the company's clinical specialist, under physician's guidance.

### Study design and data collection

This was a retrospective single-center observational study. Institutional Review Board (IRB) approval was secured from the institution (IRB #00146998) before initiating the study. Data points were retrospectively collected based on chart review and extracted from the institution's electronic medical records database. These were collected post-SCS implant at 7 days, 3 weeks, 6 weeks, 3 months and 12 months average. Data was cross checked for accuracy by the authors using governmental prescription monitoring program online database and the device manufacturer database.

### Outcome measures

Outcome measures extracted and analyzed included domains such as pain relief measured via the numeric rating scale (NRS) and disability/function evaluated using the Oswestry Disability Index (ODI). Patients were asked to report their self-improvement. It is standard of our practice to ask the patient's overall subjective improvement in pain, in a percent (%) scale, where 0% is no improvement and 100% improvement equals to complete resolution. This concept is similar to the self-reported measure of patient global impression of change. Patients were asked by staff or physician to provide a number within the scale at each visit. HCU trends were gauged by the number of emergency department (ED) visits, outpatient visits for interventional procedures, and opioid utilization measured in morphine milliequivalents (MME). Data points were analyzed for 12-month pre-and post-implant periods. These outcomes were analyzed individually in each group, and then head-to-head compared between groups.

### Statistics

Data management and statistical analyses were conducted using SAS software (version 9.4) [Copyright (c) 2002–2012 by SAS Institute Inc., Cary, NC, USA, All Rights Reserved]. Categorical variables were summarized using percentages, while continuous variables were summarized using means and standard deviation. To compare responses before and after SCS implantation, Paired *T*-test was employed for variables such as morphine milliequivalents (MME), ED visits, and outpatient procedure visits. Furthermore, comparisons between subjects with and without a history of lumbar spine surgery were performed using Independent Two-Sample *T*-Tests, evaluating baseline minus 12-month differences for self-reported pain improvement, as well as MME, ED visits, and outpatient visits. Descriptive statistics and comprehensive statistical analysis were employed as above to determine statistical significance (*p*-value <0.05) and ascertain the minimally clinically important difference (MCID). Our study aimed to determine the proportion of our sample achieving MCID in pain, disability, and opioid reduction, using the NRS, ODI and MME, respectively. Utilizing descriptive statistics combined with patient global assessment methods, where patients rate their perceived improvement, the MCID was determined based on the change score that corresponds to a certain level of improvement previously established in the literature. It is proposed that a 30% change from baseline scores may be considered a minimally clinically important difference or clinically meaningful improvement (24, 25). Specifically, for chronic pain patients, a 2-point reduction in NRS has been established as the MCID in pain outcomes (24). Similarly, MCID in disability outcomes is defined as a 10-point reduction in ODI (25). Furthermore, a 30% reduction in opioid dosage from baseline has been established as the MCID in chronic pain patients (26).

## Results

Our study population consisted of 160 subjects, 81 (50.6%) with a history of spinal surgery (group A) and 79 (49.4%) without spinal surgery (group B). Of these, 43.1% were males and 56.9% were females. Subjects had a mean age of 62 years and a mean body mass index (BMI) of 32. Table 1 summarizes demographic data and patient characteristics.

**Table 1:** Demographic and patient characteristics at baseline.

Characteristics at baseline	Category	*N* (%) Total = 160
Gender	Male	69 (43.1)
	Female	91 (56.9)
History of alcohol use	No	87 (54.4)
	Yes	73 (45.6)
History of tobacco use	No	100 (62.5)
	Yes	60 (37.5)
History of diabetes	No	110 (68.8)
	Yes	50 (31.3)
History of psychiatric illness	No	73 (45.6)
	Yes	87 (54.4)
History of spine surgery	No	79 (49.4)
	Yes	81(50.6)

The overall self-reported improvement in pain among all participants was 67.5%, with group A (surgical) mean improvement of 66.8% and 68.3% for group B (nonsurgical). Meaningful clinical important difference (MCID) in pain relief calculated based on the NRS was achieved in 37% of subjects in group A and 39% of subjects in group B. Furthermore, MCID in disability calculated based on the ODI was achieved in 45% of subjects in group A, and 42% of group B participants (24, 25). Interestingly, MCID in pain relief (NRS) and disability (ODI) was near equivalent between cohorts. Both groups had a reduction in pain and disability scores from the baseline, however, group B did not reach statistically significant reduction in disability. There was a statistically significant reduction in outpatient visits for interventional procedures for both groups individually, and only group A demonstrated a statistically significant reduction in ED visits from baseline. Interestingly, there was no statistically significant change in opioid use in either group. However, when subjects were analyzed as a combined cohort (surgical and nonsurgical), there was a statistically significant reduction (*p* < 0.0001) in opioid use, as measured by changes in MME with a mean decrease of 24.5 MME overall and a mean of 78.2% dose reduction with 91.5% reaching the MCID of a 30% dose decrease (26). Table 2 summarizes outcome changes in each cohort.

**Table 2:** The outcome changes from baseline to 12-month follow-up in group A and group B.

Group A (surgical history)	Outcome	Baseline (mean +/- std 95% CI)	12-month follow-up (mean +/- std 95% CI)	*p*-value	% subjects reached MCID
	NRS	5.63 +/- 1.8 (5.2–6.0)	4.53 +/- 1.7 (4.2–4.9)	0.0005*	37%
	ODI	44.86 +/- 14.7 (41.5–48.2)	38.10 +/- 14.5 (34.6–41.6)	0.009*	45%
	ED	0.15 +/- 0.5 (0.1–0.3)	0.04 +/- 0.2 (−0.0–0.1)	0.04*	n/a
	MME	36.29 +/- 48 (25.7–46.9)	25.27 +/- 30.3 (18.6–32)	0.098	30%
	Outpatient visits for interventional treatment of pain	1.47 +/- 1.8 (1.1–1.9)	0.28 +/- 0.6 (0.2–0.4)	0.0001*	n/a
Group B (nonsurgical)	Outcome	Baseline (mean)	12-month follow-up (mean)	*p*-value	
	NRS	5.53 +/- 2.2 (5.1–6.0)	4.45 +/- 1.9 (4.0–4.9)	0.0007*	39%
	ODI	42.21 +/- 15.8 (38.3–46.1)	38.07 +/- 15.7 (34.3–41.9)	0.22	42%
	ED visits	0.09 +/- 0.4 (0.0–0.2)	0.03 +/- 0.2 (−0.0–0.1)	0.13	n/a
	MME	27.78 +/- 32.4 (20.5–35.1)	33.63 +/- 33.2 (26.2–41.1)	0.28	23%
	Outpatient visits for interventional procedures	1.30 +/- 1.8 (0.9–1.7)	0.27 +/- 0.6 (0.1–0.4)	<0.0001*	n/a

* represents statistically significant values.

To answer our research question: “Does a history of spinal surgery affect functional outcomes and HCU trends following 10kHz SCS therapy?”, we further explored our analysis and compared pain and disability outcomes and HCU trends between the surgical and nonsurgical groups. There was no statistical significance in pain and disability outcomes between the two groups. HCU trends measured by the mean number of ED and outpatient visits for interventional pain procedures were not statistically significant between the surgical and non-surgical groups, however opioid utilization was marginally different between the two cohorts (*p* < 0.049). Table 3 summarizes these findings. There were no significant adverse events reported.

**Table 3:** Comparison of outcome changes between groups A (surgical) and B (nonsurgical).

Comparison (surgical A vs. nonsurgical B)	Outcome	*p*-value
	NRS	0.97
	ODI	0.52
	ED visits	0.47
	MME	0.04*
	Outpatient visits for interventional procedures	0.62

* represents statistically significant values.

## Discussion

This study found that 10 kHz SCS therapy was equally effective in providing MCID improvements in pain, disability and health care utilization, regardless of a history of spinal surgery. The overall improvement in pain among all participants was 67.5% and MCID in disability was achieved in 45% of subjects in group A, and 42% of group B participants. There was no statistical significance in pain and disability outcomes between the two groups, nor differences in HCU trends measured by ED and outpatient procedure visits. Our study uniquely reports findings on one of the largest cohorts of real-world 10 kHz SCS data published to date analyzing pain, function, and HCU trends collectively, in patients with and without a history of lumbar spine surgery.

SCS has been used for decades to manage CLBP and leg pain in the setting of prior lumbar spine surgery (6, 27–29). More recently, there has been a growing body of literature to support this therapy, in particular 10 kHz SCS in NSRBP treatment (12, 19, 30–32). Society guidelines, systematic reviews and meta-analysis support these findings (9, 33–35). Different patient factors may contribute to pain and functional outcomes following SCS treatment, as such spinal surgical history is an important variable to consider. Kapural et al. found that neuropathic pain phenotype and female gender had higher odds of being responders, while higher age and depression scores independently reduce the odds of pain and functional improvement (18). Similarly, our group found in a previous study high levels of kinesiophobia and pain catastrophizing behavior in nonresponders to 10kHz SCS therapy (36). Yet, pain etiology or prior history of spine surgery was not a predictive factor (18, 36). As such, our study aimed to answer the research question if a history of spinal surgery affects functional outcomes and HCU trends following 10kHz SCS therapy.

Our study reported improvements in both groups, however, beyond statistical significance, detecting MCID is critical to understand the impact of therapeutic modalities in pain practice. It has been proposed that a 30% change from baseline may be considered clinically meaningful improvement. In particular for chronic pain subjects, a 2-point reduction in NRS has been established as MCID (24, 25). We found that pain relief was statistically significant in each cohort and non-different between cohorts. Importantly, near equal MCID in pain relief between the surgical and nonsurgical group was seen. MCID in disability outcomes is defined as a 10-point reduction in ODI (25). Our study found statistically significant improvement in disability in the surgical group alone, however MCID in disability was achieved in 45% of subjects in group A, and 42% of group B participants.

The first RCT to evaluate the efficacy of 10 kHz SCS vs. CMM for the treatment of NSRBP found significant improvements in both pain and disability in the 10 kHz SCS group compared to CMM (19). This is significant as their responder rate was similar to prior studies, which evaluated this intervention in PSPS type II patients (27–30). A recent systematic review reported that SCS provides more benefits and are cost-saving compared to CMM for patients with NSRBP (37). Our findings agree with previously published studies discussing disability outcomes following SCS (38–41). Furthermore, our study confirmed prior findings that subjects with and without a spinal surgery history showed similar improvements in pain, disability with traditional low-frequency SCS (39). Both low-frequency and high-frequency SCS can improve CLBP regardless of whether patients have had previous spine surgery (12, 39). Studies exploring HCU trends and cost-effectiveness of 10 kHz SCS, both in the surgical and nonsurgical population have been published (41–43). Subjects with CLBP are known high utilizers of healthcare resources, with most of the costs from ED visits and outpatient services, including interventional procedures (4). Our study analyzed HCU outcomes, as measured by the mean number of ED visits, outpatient visits for interventional pain procedures and opioid utilization in MME. We found a statistically significant reduction in outpatient visits for interventional procedures for both groups individually, and only group A demonstrated a statistically significant reduction in ED visits from baseline. We hypothesize this may be related to the nonsurgical group reporting lower visits at baseline, compared to the surgical group. Interestingly, there was no statistically significant change in opioid use in either group, however, when subjects were analyzed as a combined cohort (surgical and nonsurgical subjects), there was a statistically significant reduction (*p* < 0.0001) in opioid use with a mean decrease of 24.5 MME overall and a mean of 78.2% dose reduction with 91.5% reaching the MCID of a 30% dose decrease (26). These findings corroborate prior studies on 10 kHz SCS opioid sparing effects (44–46). Our findings support the findings of previous studies that have evaluated the cost-effectiveness of 10 kHz SCS in surgical and nonsurgical patients individually (42, 43, 47–49).

## Limitations

Our study has limitations. Selection bias could be present as this was a non-blinded, non-randomized retrospective study. These factors may limit the generality and interpretation of results. We attempted to offset unintended bias by enrolling a large cohort of consecutive subjects with a broad study eligibility criterion. Outcome measures were extracted from electronic health records within a single institution, however some of the subjects may have sought and received care outside of the institution during the follow-up period. Data extraction and verification related to opiate use was optimized by cross-checking with governmental prescription monitoring databases. There was no specific protocol by any of the physicians involved in this study to reduce opioid prescription prior to SCS implantation; therefore, subjects were included regardless of their opioid status at baseline and without a predefined tapering process. Additionally, the simplified approach involving a single-center retrospective analysis of the frequency of healthcare utilization trends should not be considered equivalent to a full cost-effectiveness analysis, which was beyond the scope of this study. Our results are promising to suggest comparable efficacy of 10 kHz SCS therapy in patients with and without a history of lumbar spine surgery. However, further studies are needed, particularly with a prospective, blinded, randomized, and controlled methodology. Moreover, comprehensive cost-effectiveness analysis of SCS therapy is warranted, particularly in comparing groups with and without a history of lumbar spine surgery.

## Conclusion

CLBP is the leading cause of disability in the United States with an overall rising trajectory of healthcare expenditure. Therefore, it is of the utmost importance to evaluate strategies to reduce disability and improve HCU, particularly in high utilizers of healthcare resources, such as CLBP subjects. SCS therapy has demonstrated efficacy in reducing pain, improving function and lessening HCU. Nevertheless, different patient factors are important to consider that may play a role in such success. As such, our study aimed to analyze if a history of spinal surgery affects functional outcomes and HCU trends following 10 kHz SCS therapy. This is the first study to analyze pain, disability and HCU trends comparing surgical and non-surgical populations following 10 kHz SCS therapy. There was no statistical difference in pain and disability outcomes between the surgical and nonsurgical groups. HCU trends measured by the mean number of ED and outpatient visits for interventional pain procedures were not statistically different between the surgical and non-surgical groups. The results may suggest that a history of spinal surgery might not significantly impact the effectiveness of 10 kHz SCS therapy in terms of meaningful clinical importance in pain relief, functional improvement, and healthcare utilization. Further high-quality prospective and randomized clinical studies are needed to thoroughly answer this clinical question.

## Data Availability

The raw data supporting the conclusions of this article will be made available by the authors, without undue reservation.

## References

[1] Spears CA Hodges SE Kiyani M Yang Z Edwards RM Musick A Health care resource utilization and management of chronic, refractory low back pain in the United States. Spine. (2020) 45(20):E1333–41. 10.1097/BRS.0000000000003572 32453242 PMC8875812

[2] Hartvigsen J Hancock MJ Kongsted A Louw Q Ferreira M Genevay S What low back pain is and why we need to pay attention. The Lancet. (2018) 391(10137):2356–67. 10.1016/S0140-6736(18)30480-X 29573870

[3] Vos T Flaxman AD Naghavi M Lozano R Michaud C Ezzati M Years lived with disability (YLDs) for 1160 sequelae of 289 diseases and injuries 1990–2010: a systematic analysis for the Global Burden of Disease Study 2010. The Lancet. (2012) 380(9859):2163–96. 10.1016/S0140-6736(12)61729-2 23245607 PMC6350784

[4] Kirsch EP Yang LZ Lee HJ Parente B Lad SP. Healthcare resource utilization for chronic low back pain among high-utilizers. Spine J. (2024) 24(4):601–16. 10.1016/j.spinee.2023.11.017 38081464 PMC13359336

[5] Elsamadicy AA Farber SH Yang S Hussaini SMQ Murphy KR Sergesketter A Impact of insurance provider on overall costs in failed back surgery syndrome: a cost study of 122,827 patients. Neuromodulation. (2017) 20(4):354–60. 10.1111/ner.12584 28322477 PMC5482408

[6] Tieppo Francio V Leavitt L Alm J Mok D Yoon BV Nazir N Healthcare utilization (HCU) reduction with high-frequency (10 kHz) spinal cord stimulation (SCS) therapy. Healthcare (Basel). (2024) 12(7):745. 10.3390/healthcare12070745 38610166 PMC11012032

[7] Kapural L Yu C Doust MW Gliner BE Vallejo R Sitzman BT Novel 10-kHz high-frequency therapy (HF10 therapy) is superior to traditional low-frequency spinal cord stimulation for the treatment of chronic back and leg pain: the SENZA-RCT randomized controlled trial. Anesthesiology. (2015) 123(4):851–60. 10.1097/ALN.0000000000000774 26218762

[8] Bicket MC Dunn RY Ahmed SU. High-Frequency spinal cord stimulation for chronic pain: pre-clinical overview and systematic review of controlled trials. Pain Med. (2016) 17(12):2326–36. 10.1093/pm/pnw156 28025366

[9] Sayed D Grider J Strand N Hagedorn JM Falowski S Lam CM The American society of pain and neuroscience (ASPN) evidence-based clinical guideline of interventional treatments for low back pain. J Pain Res. (2022) 15:2801–832. 10.2147/JPR.S378544. Erratum in: J Pain Res. 2022 15:4075-4076. 36510616 PMC9739111

[10] Kurt E Noordhof RK van Dongen R Vissers K Henssen D Engels Y. Spinal cord stimulation in failed back surgery syndrome: an integrative review of quantitative and qualitative studies. Neuromodulation. (2022) 25(5):657–70. 10.1016/j.neurom.2021.11.013 35803677

[11] Francio V T Polston KF Murphy MT Hagedorn JM Sayed D. Management of chronic and neuropathic pain with 10 kHz spinal cord stimulation technology: summary of findings from preclinical and clinical studies. Biomedicines. (2021) 9(6):644. 10.3390/biomedicines9060644 34200097 PMC8229652

[12] Patel NP Jameson J Johnson C Kloster D Calodney A Kosek P Durable responses at 24 months with high-frequency spinal cord stimulation for nonsurgical refractory back pain. J Neurosurg Spine. (2023) 40(2):229–39. 10.3171/2023.9.SPINE23504 37976509

[13] Palmer N Guan Z Chai NC. Spinal cord stimulation for failed back surgery syndrome – patient selection considerations. Transl Perioper Pain Med. (2019) 6(3):81–90. 10.31480/2330-4871/093 31687422 PMC6828157

[14] Petersen EA Schatman ME Sayed D Deer T. Persistent spinal pain syndrome: new terminology for a new era. J Pain Res. (2021) 14:1627–30. 10.2147/JPR.S320923 34135626 PMC8197591

[15] Christelis N Simpson B Russo M Stanton-Hicks M Barolat G Thomson S Persistent spinal pain syndrome: a proposal for failed back surgery syndrome and ICD-11. Pain Med. (2021) 22(4):807–18. 10.1093/pm/pnab015 33779730 PMC8058770

[16] Schug SA Lavand’homme P Barke A Korwisi B Rief W Treede RD The IASP classification of chronic pain for ICD-11: chronic postsurgical or posttraumatic pain. Pain. (2019) 160(1):45–52. 10.1097/j.pain.0000000000001413 30586070

[17] Van de Minkelis J Peene L Cohen SP Staats P Al-Kaisy A Van Boxem K Update of evidence-based interventional pain medicine according to clinical diagnoses 6. Persistent spinal pain syndrome type 2. Pain Pract. (2024) 00:1–18. 10.1111/papr.13379 38616347

[18] Kapural L Wu C Calodney A Pilitsis J Bendel M Petersen E Demographics and PainDETECT as predictors of 24-month outcomes for 10 kHz SCS in nonsurgical refractory back pain. Pain Physician. (2024) 27(3):129–39. 38506680

[19] Kapural L Jameson J Johnson C Kloster D Calodney A Kosek P Treatment of nonsurgical refractory back pain with high-frequency spinal cord stimulation at 10 kHz: 12-month results of a pragmatic, multicenter, randomized controlled trial. J Neurosurg Spine. (2022) 37(2):188–99. 10.3171/2021.12.SPINE211301 35148512

[20] Petersen EA Stauss TG Scowcroft JA Brooks ES White JL Sills SM Effect of high-frequency (10-kHz) spinal cord stimulation in patients with painful diabetic neuropathy: a randomized clinical trial. JAMA Neurol. (2021) 78(6):687–98. 10.1001/jamaneurol.2021.0538 33818600 PMC8022268

[21] Manchikanti L Abdi S Atluri S Benyamin RM Boswell MV Buenaventura RM An update of comprehensive evidence-based guidelines for interventional techniques in chronic spinal pain. Part II: guidance and recommendations. Pain Physician. (2013) 16(2 Suppl):S49–S283. 10.36076/ppj.2013/16/s49 23615883

[22] Qaseem A Wilt TJ McLean RM Forciea MA Denberg TD Barry MJ Noninvasive treatments for acute, subacute, and chronic low back pain: a clinical practice guideline from the American college of physicians. Ann Intern Med. (2017) 166(7):514–30. 10.7326/M16-2367 28192789

[23] Eckermann JM Pilitsis JG Vannaboutathong C Wagner BJ Province-Azalde R Bendel MA. Systematic literature review of spinal cord stimulation in patients with chronic back pain without prior spine surgery. Neuromodulation. (2022) 25(5):648–56. 10.1111/ner.13519 34407288

[24] Farrar JT Young JP Jr LaMoreaux L Werth JL Poole MR. Clinical importance of changes in chronic pain intensity measured on an 11-point numerical pain rating scale. Pain. (2001) 94(2):149–58. 10.1016/S0304-3959(01)00349-9 11690728

[25] Ostelo RW Deyo RA Stratford P Waddell G Croft P Von Korff M Interpreting change scores for pain and functional status in low back pain: towards international consensus regarding minimal important change. Spine. (2008) 33(1):90–4. 10.1097/BRS.0b013e31815e3a10 18165753

[26] Goudman L Smedt A Forget P Moens M. Determining the minimal clinical important difference for medication quantification scale III and morphine milligram equivalents in patients with failed back surgery syndrome. J Clin Med. (2020) 9(11):3747. 10.3390/jcm9113747 33233343 PMC7700681

[27] Kapural L Peterson E Provenzano DA Staats P. Clinical evidence for spinal cord stimulation for failed back surgery syndrome (FBSS): systematic review. Spine. (2017) 42(Suppl 14):S61–6. 10.1097/BRS.0000000000002213 28441313

[28] Van Buyten JP Al-Kaisy A Smet I Palmisani S Smith T. High-frequency spinal cord stimulation for the treatment of chronic back pain patients: results of a prospective multicenter European clinical study. Neuromodulation. (2013) 16(1):59–66. 10.1111/ner.12006 23199157

[29] Kapural L Yu C Doust MW Gliner BE Vallejo R Sitzman BT Comparison of 10-kHz high-frequency and traditional low-frequency spinal cord stimulation for the treatment of chronic back and leg pain: 24-month results from a multicenter, randomized, controlled pivotal trial. Neurosurgery. (2016) 79(5):667–77. 10.1227/NEU.0000000000001418 27584814 PMC5058646

[30] Al-Kaisy A Palmisani S Smith TE Pang D Lam K Burgoyne W 10 Khz high-frequency spinal cord stimulation for chronic axial low back pain in patients with no history of spinal surgery: a preliminary, prospective, open label and proof-of-concept study. Neuromodulation. (2017) 20:63–70. 10.1111/ner.12563 28025843

[31] Patel N Calodney A Kapural L Province-Azalde R Lad SP Pilitsis J High-Frequency spinal cord stimulation at 10 kHz for the treatment of nonsurgical refractory back pain: design of a pragmatic, multicenter, randomized controlled trial. Pain Pract. (2021) 21(2):171–83. 10.1111/papr.12945 33463027 PMC7891432

[32] Kapural L Calodney A. Retrospective efficacy and cost-containment assessment of 10 kHz spinal cord stimulation (SCS) in non-surgical refractory back pain patients. J Pain Res. (2022) 15:3589–95. 10.2147/JPR.S373873 36415659 PMC9676005

[33] Baranidharan G Edgar D Bretherton B Crowther T Lalkhen AG Fritz AK Efficacy and safety of 10 kHz spinal cord stimulation for the treatment of chronic pain: a systematic review and narrative synthesis of real-world retrospective studies. Biomedicines. (2021) 9(2):180. 10.3390/biomedicines9020180 33670252 PMC7918133

[34] Zheng Y Liu CW Hui Chan DX Kai Ong DW Xin Ker JR Ng WH Neurostimulation for chronic pain: a systematic review of high-quality randomized controlled trials with long-term follow-up. Neuromodulation. (2023) 26(7):1276–94. 10.1016/j.neurom.2023.05.003 37436342

[35] ElSaban M Kleppel DJ Kubrova E Martinez Alvarez GA Hussain N D’Souza RS. Physical functioning following spinal cord stimulation: a systematic review and meta-analysis. Reg Anesth Pain Med. (2023) 48(6):302–11. 10.1136/rapm-2022-104295 37080578

[36] Francio V T Alm J Leavitt L Mok D Yoon BV Nazir N Variables associated with nonresponders to high-frequency (10 kHz) spinal cord stimulation. Pain Pract. (2024) 24(4):584–99. 10.1111/papr.13328 38078593

[37] Eldabe S Nevitt S Bentley A Mekhail NA Gilligan C Billet B Network meta-analysis and economic evaluation of neurostimulation interventions for chronic non-surgical refractory back pain. Clin J Pain. (2024) 40(9):507–17. 10.1097/AJP.0000000000001223 38751011 PMC11309338

[38] Paul AR Kumar V Roth S Gooch MR Pilitsis JG. Establishing minimal clinically important difference of spinal crd stimulation therapy in post-laminectomy syndrome. Neurosurgery. (2017) 81(6):1011–5. 10.1093/neuros/nyx153 28973581

[39] Campwala Z Datta P DiMarzio M Sukul V Feustel PJ Pilitsis JG. Spinal cord stimulation to treat low back pain in patients with and without previous spine surgery. Neuromodulation. (2021) 24(8):1363–9. 10.1111/ner.13333 33314462

[40] Sabourin S Tram J Sheldon BL Pilitsis JG. Defining minimal clinically important differences in pain and disability outcomes of patients with chronic pain treated with spinal cord stimulation. J Neurosurg Spine. (2021) 35(2):243–50. 10.3171/2020.11.SPINE201431 34087802

[41] Gupta M Ray M Ladesich N Gupta A. Health-care utilization and outcomes with 10 kHz spinal cord stimulation for chronic refractory pain. J. Pain Res. (2021) 14:3675–83. 10.2147/JPR.S306126 34880672 PMC8648088

[42] Rajkumar S Yang LZ Venkatraman V Charalambous L Parente B Lee HJ Health care resource utilization of high-frequency spinal cord stimulation for treatment of chronic refractory low back pain. Neuromodulation. (2023) 26:115–23. 10.1016/j.neurom.2022.03.013 35871122

[43] Rajkumar S Venkatraman V Yang LZ Parente B Lee HJ Lad SP. Short-term health care costs of high-frequency spinal cord stimulation for the treatment of postsurgical persistent spinal pain syndrome. Neuromodulation. (2023) 26:1450–8. 10.1016/j.neurom.2023.01.016 36872148

[44] Al-Kaisy A Van Buyten JP Amirdelfan K Gliner B Caraway D Subbaroyan J Opioid-sparing effects of 10 kHz spinal cord stimulation: a review of clinical evidence. Ann N Y Acad Sci. (2020) 1462:53–64. 10.1111/nyas.14236 31578744 PMC7065058

[45] Feng H Doherty P Rotte A. Decreased opioid consumption and durable pain relief in patients treated with 10 kHz SCS: a retrospective analysis of outcomes from single-center. J Pain Res. (2021) 14:2593–600. 10.2147/JPR.S312932 34466027 PMC8403026

[46] Rupp A Francio VT Hagedorn JM Deer T Sayed D. The impact of spinal cord stimulation on opioid utilization in failed back surgery syndrome and surgery naive patients. Interv. Pain Med. (2022) 1:100148. 10.1016/j.inpm.2022.100148 39238856 PMC11372956

[47] DiBenedetto DJ Wawrzyniak KM Schatman ME Kulich RJ Finkelman M. 10 Khz spinal cord stimulation: a retrospective analysis of real-world data from a community-based, interdisciplinary pain facility. J Pain Res. (2018) 11:2929–41. 10.2147/JPR.S188795 Erratum in: J Pain Res. 2019 12:543-544. 30538532 PMC6251433

[48] Niyomsri S Duarte RV Eldabe S Fiore G Kopell BH McNicol E A systematic review of economic evaluations reporting the cost-effectiveness of spinal cord stimulation. Value Health. (2020) 23:656–65. 10.1016/j.jval.2020.02.005 32389232

[49] Figueroa C Hadanny A Kroll K DiMarzio M Ahktar K Gillogly M Does neuromodulation reduce chronic pain patient emergency department utilization? Neurosurgery. (2021) 90:131–9. 10.1227/NEU.0000000000001754 34982880

